# Activation of the Bile Acid Pathway and No Observed Antimicrobial Peptide Sequences in the Skin of a Poison Frog

**DOI:** 10.1534/g3.118.200880

**Published:** 2019-01-03

**Authors:** Megan L. Civitello, Robert Denton, Michael A. Zasloff, John H. Malone

**Affiliations:** * Institute of Systems Genomics, Department of Molecular and Cell Biology, University of Connecticut, Storrs, Connecticut 06269; † Georgetown University School of Medicine, MedStar Georgetown Transplant Institute, Washington D.C. 20057

**Keywords:** Anti-microbial peptides, defensive secretions, phylogenetic history, bile acid pathway

## Abstract

The skin secretions of many frogs have genetically-encoded, endogenous antimicrobial peptides (AMPs). Other species, especially aposematic poison frogs, secrete exogenously derived alkaloids that serve as potent defense molecules. The origins of these defense systems are not clear, but a novel bile-acid derived metabolite, tauromantellic acid, was recently discovered and shown to be endogenous in poison frogs (*Mantella*, *Dendrobates*, and *Epipedobates*). These observations raise questions about the evolutionary history of AMP genetic elements, the mechanism and function of tauromatellic acid production, and links between these systems. To understand the diversity and expression of AMPs among frogs, we assembled skin transcriptomes of 13 species across the anuran phylogeny. Our analyses revealed a diversity of AMPs and AMP expression levels across the phylogenetic history of frogs, but no observations of AMPs in *Mantella*. We examined genes expressed in the bile-acid metabolic pathway and found that *CYP7A1* (Cytochrome P450), *BAAT* (bile acid-CoA: amino acid N-acyltransferase), and *AMACR* (alpha-methylacyl-CoA racemase) were highly expressed in the skin of *M. betsileo* and either lowly expressed or absent in other frog species. In particular, *CYP7A1* catalyzes the first reaction in the cholesterol catabolic pathway and is the rate-limiting step in regulation of bile acid synthesis, suggesting unique activation of the bile acid pathway in *Mantella* skin. The activation of the bile acid pathway in the skin of *Mantella* and the lack of observed AMPs fuel new questions about the evolution of defense compounds and the ectopic expression of the bile-acid pathway.

Amphibians represent an important evolutionary transition from aquatic to terrestrial life, and the unique constraints of their life history are reflected in their skin tissue. Amphibian skin acts as the major respiratory organ for most amphibian taxa and undergoes dramatic structural and transcriptional changes during metamorphosis, which, in part, prepares the skin for production of anti-predator and protective elements (Huang *et al.* 2016).

Protective elements in amphibian skin include compounds that are anti-predatory (*i.e.*, toxins; Daly *et al.* 1987; Roelants *et al.* 2013) and compounds that provide immunological and antimicrobial functions (König *et al.* 2015). Small molecular toxins, such as alkaloids, are abundant in the skin of Neotropical and Madagascar poison dart frogs (Daly *et al.* 1997; Clark *et al.* 2012; König *et al.* 2015; Moskowitz *et al.* 2018). These molecules are exogenous, acquired from eating arthropods, especially ants and termites, and later sequestered in skin glands to mediate defense (Daly *et al.* 1987, 2005; Clark *et al.* 2005).

While alkaloids are exogenously acquired from diet, other compounds are endogenously encoded in the genome and provide immunological and antimicrobial functions to combat bacteria, fungi, protozoa, and viruses. Antibodies and T-lymphocyte-mediated responses constitute the majority of the adaptive immune defense, while the innate immune system is a composite of macrophages, neutrophils, natural killer cells, and antimicrobial peptides (AMPs) that function in a nonspecific manner to combat foreign infection (Simmaco *et al.* 1998; Zasloff 1987; Simmaco *et al.* 1993; Rollins-Smith *et al.* 2005; Simmaco *et al.* 2009; Conlon 2011; Calhoun *et al.* 2016; Ladram and Nicolas 2016; Matthijs *et al.* 2017).

AMPs have been one of the most well-studied and important components of the innate immune system in amphibians and have been a major focus for the development of therapeutics for humans (Zasloff 2002a, 2002b). These peptides have specific cationic and hydrophobic regions that allow them to target bacteria, fungi, enveloped viruses, and some cancerous cells for lysis (Reddy *et al.* 2004). Several amphibian taxa have independently acquired the genes to produce skin secreted peptide arsenals and these arsenals are diverse across the phylogeny of frogs (Roelants *et al.* 2013; König *et al.* 2015; Xu and Lai 2015). The question remains as to how AMPs originate and the functional diversification among defense arsenals in multiple taxa; though at least in *Xenopus*, some AMPs appear to be derived from a gene cluster that originally had a gastrointestinal hormone function (Roelants *et al.* 2013).

Another open question is how interactions between different classes of compounds and chemical communities in amphibian skin accomplish diverse protective functions. However, investigating this question is made difficult by the rate at which new compounds are still being discovered, observations of AMPs in some but not other taxa, and the small fraction of species that have been studied (König *et al.* 2015). Recently, a new bile acid derived compound, named tauromantellic acid (TMA), was discovered from the skin of Madagascar poison frogs in the genus *Mantella* and poison dart frogs in the genera *Dendrobates* and *Epipedobates* (Clark *et al.* 2012). This was the first example of a bile acid found within the skin secretion of any frog (Clark *et al.* 2012). Since bile acids normally act as surfactants to aid intestinal digestion, Clark *et al.* (2012) hypothesized that tauromantellic acid could potentially shield frogs from their own toxicity via the creation of micelles that sequester or aid in the uptake of alkaloids (Clark *et al.* 2012). However, the ring structure of TMA shares striking similarity to that of the broad spectrum antimicrobial aminosterol, squalamine, previously isolated from many tissues of the dogfish shark (*Squalus acanthias*), including the liver and gallbladder (Moore *et al.* 1993; Rao *et al.* 2000) and the white blood cells of the sea lamprey (*Petromyzon marinus*; Yun and Li 2007), raising questions as to whether TMA could play a role in antimicrobial functions.

Squalamine is a potent, broad spectrum antimicrobial in which a polyamine (spermidine) is coupled to the steroid ring scaffold of a bile acid (Moore *et al.*, 1993). Although TMA has not been assayed for antimicrobial activity, bile acids of similar structure are known to exhibit weak activity (Sannasiddappa *et al.* 2017), about 1000-fold lower than frog skin AMPs, and we expect TMA itself will not exhibit potency comparable to frog skin AMPs. However, the union of an anionic bile acid with a cationic polyamine is sufficient to create an amphipathic compound with potent antibacterial activity (Jones *et al.* 1996; Savage *et al.* 2002; Shu *et al.* 2002; Tessema *et al.* 2013).

The diversity of AMPs and other compounds in frog skin raises new questions about the evolutionary history of AMPs, their relationship with other compounds, and their collective functions. To begin to answer these questions, we compared gene expression in the skin of thirteen frog species, including a newly generated skin transcriptome for *Mantella betsileo*. We used the Database of Anuran Defense Peptides (Novković *et al.* 2012) to identify the presence of AMPs in frog taxa spanning the anuran phylogeny. Additionally, we analyzed expression of genes in the primary bile acid biosynthesis pathway in order to look for the functional signals of tauromantellic acid in other frogs and we tested the antimicrobial activity of MA coupled with spermine. Collectively, we show that AMPs are widespread in the frogs we studied, with a likely absence in *Mantella*, that genes needed for the bile acid pathway are uniquely expressed in *Mantella* skin, and that there is significant anti-microbial activity for MA when it is coupled to a spermine. Taken together, our results show a correlation between a possible absence of AMPs and activation of the bile acid pathway in the skin of *Mantella*.

## Materials and Methods

### Sequencing and Transcriptome Assembly

We harvested total RNA from the dorsal and ventral skin of five captive bred *Mantella betsileo* that were anesthetized by carbon dioxide according to Georgetown Institutional Animal Care and Use Protocol No. 2016-1351. We transferred the skin collections to a tube of All-Protect reagent and extracted RNA using a Qiagen RNA kit using manufacturer’s protocols (Qiagen, Germany). The mRNA from *M. betsileo* skin was enriched using oligo-dT primers and fragmented into approximately 200 nt fragments by alkaline hydrolysis. The cDNA library was generated via random hexamer priming and sequenced on an Illumina HiSeq 2000 sequencer to achieve paired-end 90 base pair sequences. We assessed read quality with a FastQC Report v.0.11.2, and trimmed reads using Sickle v.1.33 with a quality score of 30 and a minimum sequence length of 45 base pairs. Approximately 54 million raw reads were generated and ∼47 million reads passed quality filtering after trimming with Sickle v.1.33 (Joshi and Fass 2011). Following trimming, we assembled the transcriptome of *M. betsileo* using Trinity v.2.4.0 (Grabherr *et al.* 2011; Haas *et al.* 2013) using a minimum contig length of 300. We assessed the continuity of the assembly by the contig N50 statistic and measured read representation by aligning reads back to the transcriptome with Bowtie2 v.2.3.1 (Langmead and Salzberg 2012) to identify proper pairs and lone read alignments. We used BUSCO v.3 (Simão *et al.* 2015; Waterhouse *et al.* 2018) to estimate the completeness of the transcriptome based on a set of conservative Eukaryotic orthologs and Transrate v.1.0.3 (Smith-Unna *et al.* 2016) to evaluate the accuracy and completeness of the *de novo* assembly.

### Gene Identification and Expression Levels

To identify candidate-coding regions, we used TransDecoder v.3.0.0 (Haas and Papanicolaou 2016) with default parameters along with BlastP v.2.2.29+ and Pfam v.31.0 (Finn *et al.* 2016). We used Blastx v.2.2.29+ to search for matches for each transcript against the Swissprot database (The UniProt Consortium 2017) and used an e-value of 1e-20 to identify matches. In this manner, we were able to search the peptides identified by TransDecoder as well as the entire set of transcripts. To determine if transcripts were antimicrobial peptide precursors, we conducted BLAST searches to the Database of Anuran Defense Peptides (DADP; Novković *et al.* 2012). The DADP is a manually created database of all known antimicrobial amphibian peptides and when identified, contains both the more conserved signal and the more divergent, bioactive domain sequences. We also repeated the BLAST using a less stringent value of 1e-5 to search for more loosely matching AMPs that might exist but could be difficult to detect in *M. betsileo* due to sequence divergence. Additionally, we used HMMER v. 3.2.1 (Eddy 2009) with an e-value of 1e-5 to search assembled transcripts for matches to the Pfam database (Finn *et al.* 2016). The Pfam database is a collection of protein families, sourced from UniProtKB sequences, represented by multiple sequence alignments and hidden Markov models (HMMs), which we used as another method to detect the presence of antimicrobial peptide domains in the frog skin transcriptomes. We used RSEM v.1.3.0 (Li and Dewey 2011) to identify gene expression levels and assigned functional annotations using Trinotate v.3.0.2 (Haas *et al.* 2013) with default parameters. Additionally, we extracted Gene Ontology assignments using GOseq with Trinotate, which categorically identified genes with a standardized molecular, biological, or cellular function. We used R v.3.3.1 (R Core Team 2017) to generate a normalized TPM Expression Histogram of genes based on their relative categorical ranking of high, middle, or low expression value, which we determined by calculating the 25% and 75% quartiles of the entire gene data set and separating the genes into their respective expression ranges.

### Comparison of AMP Expression

We downloaded RNA-Seq data from the NCBI Short Read Archive database and selected 12 frog skin datasets that span the anuran phylogeny (Table S1). We used the raw reads for each dataset and ran Sickle, Trinity, Blast, RSEM, and HMMER using the same parameters as the *M. betsileo* dataset.

To compare expression levels between species, we used two methods of normalization. First, we created a rank order for genes in each transcriptome based on raw counts such that the lowest expressed gene was ranked 1. We then identified the location of AMPs within the rank ordered list and calculated the AMP expression percentile by dividing the ranked order number of each AMP by the total number of genes within the transcriptomes, multiplied by 100 to obtain a percentage. Thus, the highest expressed gene had an expression percentile of 100% because its ranked order number within the list was equal to the total number of genes in the transcriptome. Second, we identified common gene names among all 13 species and created a matrix of these genes and relative raw counts in each frog species. We then used edgeR v.3.6 to create a normalized expression matrix of genes across all species by correcting for library size. We converted the normalized expression counts to percentages and calculated the percentile expression of each gene within the bile acid pathway. Additionally, we generated a phylogeny of all 13 species using the Time Tree of Life (Hedges *et al.* 2015) to test for a phylogenetic signal of AMP expression (Ives *et al.* 2007). Two species were not available in the Time Tree of Life database (*Atelopus glyphus* and *Craugastor fitzingeri*), so we substituted the two most closely related species (*Atelopus zeteki* and *Craugastor longirostris*, respectively).

### Comparison of Primary Bile Acid Biosynthesis Pathway

We used the KEGG Automatic Annotation Server (KAAS; Moriya *et al.* 2007) to annotate genes found in the Primary Bile Acid Pathway in order to identify potential differences between species that may reflect functional changes. We used the identified genes from our transcriptome assembly in conjunction with the normalized percentile expression values to map genes in the primary bile acid pathway proportionalized by expression level using Cytoscape v. 3.6.0 (Shannon *et al.* 2003).

### Anti-microbial Assays

Antimicrobial activity assays of Sm dihydro-MA, ampicillin, squalamine, and MSI-1436 were carried according to procedures in Moore *et al.* (1993). The minimal inhibitory concentration (MIC) was determined by incubating logarithmic-phase organisms (about 10^6^ colony forming units/ml) in 0.5 strength trypticase soy broth at 37° for 18-24 hr in the presence of various concentrations of antimicrobial. The MIC is the concentration of antimicrobial where visible growth was inhibited.

### Data Availability

The skin transcriptome assembly and raw sequence data for *Mantella betsileo* can be found at the Gene Expression Omnibus under accession GSE61355. This Transcriptome Shotgun Assembly project has been deposited at DDBJ/EMBL/GenBank under the accession GGTL00000000. The version described in this paper is the first version, GGTL01000000. Supplemental material available at Figshare: https://doi.org/10.25387/g3.7543436.

## Results

To understand the variation in expression of AMPs and other genes expressed in the skin of frogs, we downloaded publicly available RNA-Seq data for 12 anuran species and generated a new transcriptome from *Mantella betsileo*. The publicly available data were chosen because they represented a deep sample of anuran phylogenetic history that we could use to answer questions about the evolution and diversification of AMPs and other genes expressed in frog skin compared to *Mantella*. We chose to produce RNA-Seq data for *Mantella betsileo* because this species was shown to produce TMA regardless of environmental factors (Clark *et al.* 2012). It is important to note that the publicly available data were produced under a variety of protocols and sequencing methods, which could produce non-biological variation. However, our questions focused on the presence of AMPs and genetic pathways related to phenotype, which are less likely to be impacted by the technical variation that could arise from extraction, library, and sequencing methods. We attempted to further guard against these potential confounding variables by assembling the skin transcriptome for each species using the same computational pipeline.

### Quality of Transcriptome Assembly for Mantella

Of the 23,892,703 total sequences, 77.5% (18,515,394) were retained as paired end reads after trimming with Sickle. The contig N50 value of the assembly was 910 and more than 82% of reads aligned back to the assembled transcriptome reference. To estimate the completeness of our *Mantella* transcriptome, we used BUSCO to look for 303 genes assumed to be ubiquitous in all eukaryotes. Of the 303 core BUSCO groups searched, 198 (65.3%) were identified as complete and single-copy BUSCOs. Only 8 BUSCO genes out of the 303 genes searched could not be found within the transcriptome, further validating the high level of completeness of the assembly. Transrate analyses identified the accuracy with which the complete set of transcripts was represented in the read data in the absence of a reference genome. Of the 18 million total fragments that successfully mapped back, 76.6% of reads aligned in the correct orientation on the same contig, which was comparable to other frog transcriptomes we analyzed (average = 71% (± 4.5 SE).

### No AMPs observed in Mantella betsileo

**Figure 1:**
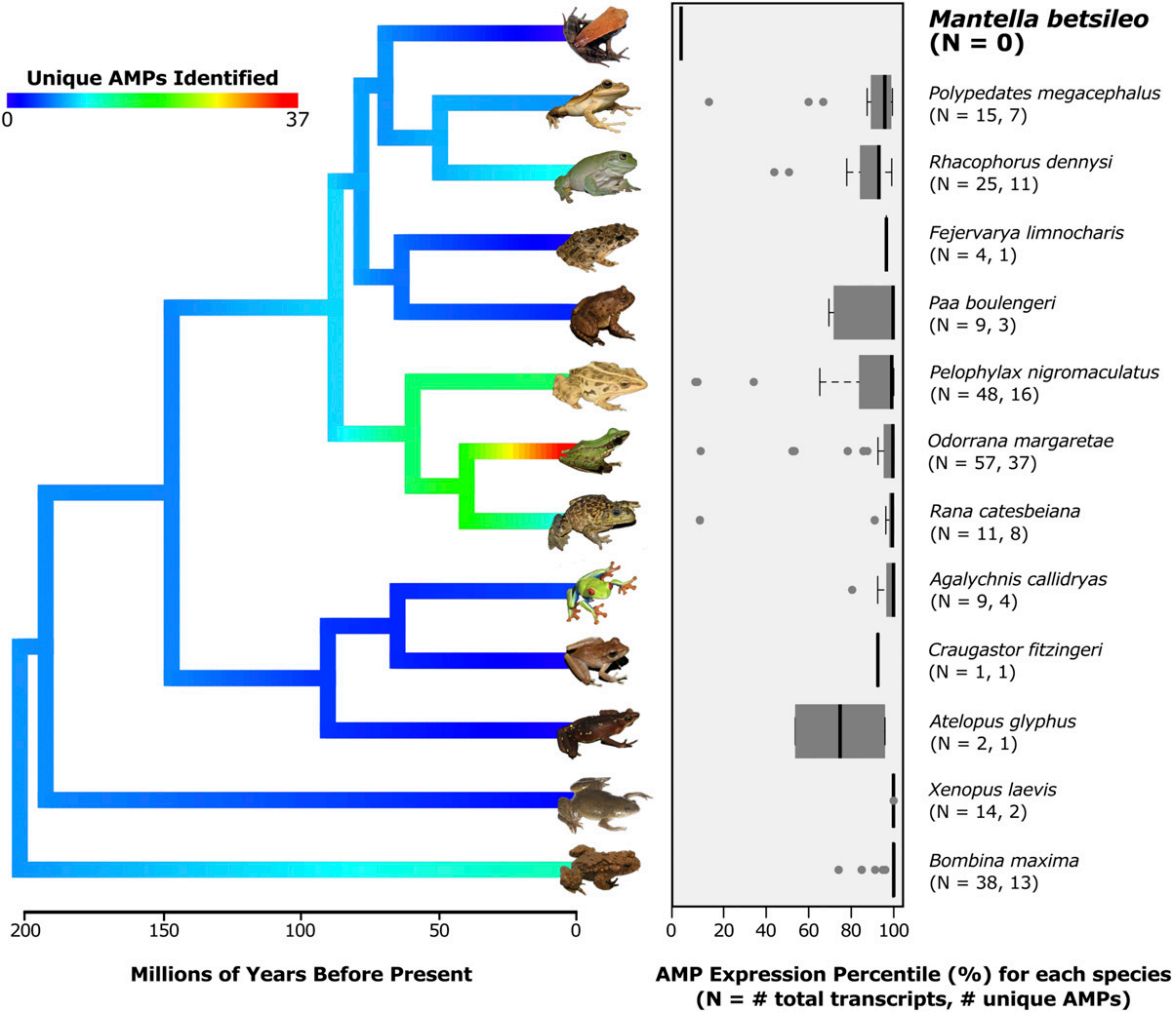
Antimicrobial peptides (AMPs) and expression levels among 13 frog skin transcriptomes. AMPs were not observed in the transcriptome of *M. betsileo*, but were highly expressed in all other frog clades. A phylogeny for thirteen frog species is shown on the left (reconstructed using TimeTree, Hedges *et al.* 2015; Kumar *et al.* 2017) and branches are colored according to the number of unique AMPs found in each taxon using phytools in R (Revell 2012). The AMP expression percentile (rank order of an AMP transcript divided by the total number of transcripts) is summarized for each taxon on the right. Sample sizes for the total number of AMP transcripts are positively correlated with the number of unique AMPs identified.

We searched for AMPs across 200 million years of frog evolutionary history and tested the hypothesis that there were changes in AMP abundance and expression among lineages. We retrieved a diversity of AMP matches by BLAST, with 74 unique AMPs in total and an average of 8.67 (± 2.96 SE) unique AMPs per lineage. *Odorrana* had the most unique AMPs (37) and *Craugastor*, *Atelopus*, and *Fejervarya* had the least (1), suggesting that there was substantial variation in the number of unique AMPs found in each lineage (Figure 1; Figure S1). However, despite this extensive variation, there was no phylogenetic signal in either the number of unique (K = 0.52, *P* = 0.47) or expression rank (K = 0.54, *P* = 0.338) for these AMPs.

AMP expression level was on average at the 92.0 (± 1.11 SE) rank percentile, revealing that AMP precursors were among the most highly expressed genes in frog skin (Figure 1). The two highest numbers of AMPs were found in *Pelophylax* and *Odorrana*, which are relatively closely related; however, there were several AMPs expressed at low levels in Ranid lineages as well as the two species most closely related to *Mantella*.

In contrast to all other frogs, we did not observe AMP sequences in the skin transcriptome of *Mantella betsileo* using BLAST against the DADP or Swissprot, or HMMER against the Pfam protein domain database (Figure 1; Figure S1; Table S2). AMPs are not annotated in the DADP or Pfam for *Mantella*; thus, the lack of annotation could explain the lack of observed AMPs even though they may be present in *Mantella*. AMP sequences are typically divergent (König *et al.* 2015) and there is not a single shared amino acid among all AMP sequences for either the more conserved signal sequence or the more rapidly evolving bioactive region (Figure S2). However, we did observe AMPs in the transcriptomes for six other species that were not known to previously have AMPs (Table 1; Table S2**)**. For these six, three species (*Atelopus glyphus*, *Craugastor fitzingeri*, and *Fejervarya limnocharis*) had highly expressed transcripts that matched to Buforin-II, a sequence that is derived from Histone 2A, and experimentally demonstrated to have antimicrobial activity (Park *et al.* 1996; 2000). Furthermore, all observed BLAST hits from the DADP to Buforin-II contained the AMP variant sequence “TRSSRAGLQFPVGRVHRLLRK”, while the remaining BLAST hits from the Swissprot Database to Histone 2A did not contain the antimicrobial bioactive sequence referenced above. The other three species (*Paa boulengeri*, *Polypedates megacephalus*, *Rhacophorus dennysi*), had sequences that matched a diversity of AMP classes (Figure S1).

**Table 1:** AMP hits to number of annotations in the Database of Anuran Defense Peptides (DADP). The underlined species are those not present in the DADP, but with hits to AMPs. The right-most column indicates the number of AMPs listed in the DADP for each species and genus. We obtained hits for AMPs in every other species not found in the database except for *Mantella*

Species	N50	AMP Hits	AMPs in Database (species: genera)
***Mantella betsileo***	**901**	**0**	**0:0**
*Polypedates megacephalus*	946	15	0:0
*Rhacophorus dennysi*	1846	25	0:2
*Fejervarya limnocharis*	900	4	0:3
*Paa boulengeri*	2030	9	0:0
*Pelophylax nigromaculatus*	918	48	5:77
*Odorrana margaretae*	1296	57	26:1148
*Rana catesbeiana*	641	11	46:358
*Agalychnis callidryas*	1630	9	6:12
*Craugastor fitzingeri*	1567	1	0:0
*Atelopus glyphus*	1055	2	0:0
*Xenopus laevis*	2045	14	15:59
*Bombina maxima*	1019	38	219:243

Second, since AMPs are among the most highly expressed genes in frog skin, we examined the most highly expressed genes across all 13 species. This approach allowed us to determine if any of the unidentified transcripts in *Mantella* with high levels of expression were related to AMP or defense function. Across all 13 species, we found that the most highly expressed genes matched to keratin and ribosomal proteins. The important distinction was the presence of AMPs in the upper expression threshold for all other species except *M. betsileo*. An alternative explanation for the lack of AMPs in *Mantella* is that we may not have had enough depth of sequencing to detect AMPs in the skin transcriptome. However, as demonstrated, AMPs are among the most highly expressed sequences and thus are more easily identified in RNA-Seq data compared to lower expressed genes (Mortazavi *et al.* 2008; Malone and Oliver 2011; Conlon 2011; Conesa *et al.* 2016).

Taken together, we observed a diversity of AMPs across nearly 200 million years of anuran evolution, but AMPs were not observed in *M. betsileo*. Our lack of observations of AMP sequences in *Mantella* suggests either that *Mantella* does not have AMPs, or if present, *Mantella* have AMPs that are so divergent that our methods cannot detect them.

### Primary Bile Acid Biosynthesis Pathway Functionality in Frog Skin

The lack of AMPs observed in *Mantella* is notable given that AMPs contribute to immunity in amphibians, are considered prominent components of most frog skin secretions, and are evolutionarily widespread (Conlon 2011; König *et al.* 2015; Figure 1; Figure S1). Recently, *Mantella* and other poison frogs were discovered to secrete a novel metabolite named tauromantellic acid. TMA has structural similarity to a bile acid, a metabolite synthesized by the liver and employed to function in the emulsification of fats and oils. Given that tauromantellic acid is endogenous and likely derived from cholesterol, we searched for the presence of genes active in the bile acid synthesis pathway and examined expression variation among species by normalizing expression data for these genes across all 13 frog skin transcriptomes.

**Figure 2:**
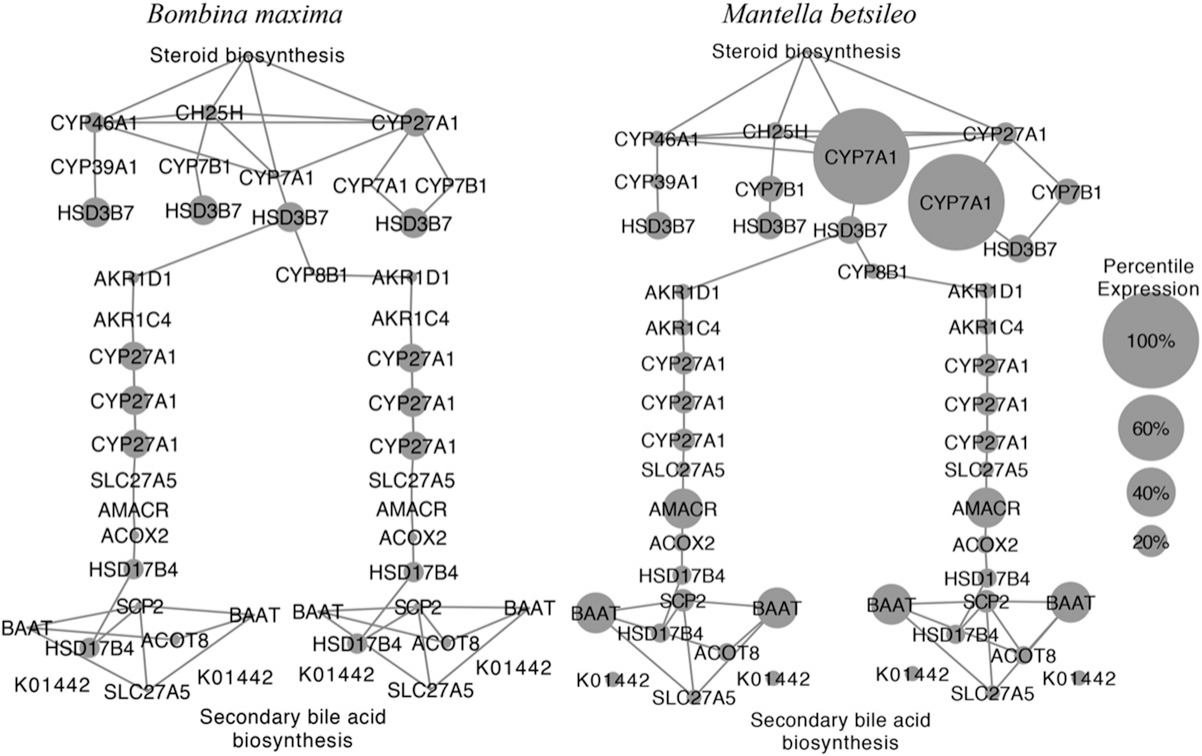
Comparison of genes involved in the primary bile acid biosynthesis pathway in *Mantella* compared to *Bombina*. Gene pathway members were identified in each transcriptome using the KEGG Automatic Annotation Server (KAAS). The raw expression counts for each gene identified on the pathway were obtained from RSEM and used to generate a normalized expression matrix across all species. Pictured is a painted network of percentile gene expression values for a representative species (*B. maxima*) in comparison to *M. betsileo* generated using Cytoscape v. 3.6.0. The key difference is the large expression value for *CYP7A1*, a gene critical for the rate limiting step of bile acid production, in *Mantella* compared to all other frog species as represented by *B. maxima. BAAT* (a gene necessary for bile secretion) and *AMACR* (a gene necessary for the degradation of methyl-branched fatty acids) are also more highly expressed in *Mantella* compared to all other species. Together, the presence and abundance in expression of *CYP7A1*, *BAAT*, and *AMACR* may suggest functionality of the bile acid pathway in the skin secretions of *M. betsileo*.

We found evidence of expression for an average of 70.4% (± 3.64 SE) of the 18 total pathway genes across the other 12 frog species; however, *M. betsileo* was the only species to have high expression for *CYP7A1*, a gene critical to the function of the bile acid pathway (Figure 2). *CYP7A1* had a rank percentile expression of 96% in *M. betsileo* *vs.* 4% in *Odorrana margaretae*, the only other species to have any evidence of expression for this gene. *CYP7A1* catalyzes the first reaction in the cholesterol catabolic pathway in the liver and is the rate-limiting step in regulation of bile acid synthesis (Hubacek and Bobkova 2006). The presence of this gene determines the functionality of the primary bile acid biosynthesis pathway and thus suggests activity in the skin of *M. betsileo*.

We observed higher expression in *Mantella* for two other genes normally expressed in liver and involved in bile acid synthesis. *BAAT* was highly expressed exclusively in *M. betsileo*. The average *BAAT* expression percentile in all other 12 species was 5.82% (± 3.60 SE) as compared with the 30.2% expression percentile observed in *Mantella*. Additionally, another gene (*AMACR*) had an average expression percentile of 5.89% (± 1.57 SE) across all other 12 species as compared to an expression percentile of 29.4% found in *Mantella*. This gene encodes a racemase essential for bile acid synthesis and conversion of pristanoyl-CoA and C27-bile acyl-CoAs to their (S)-stereoisomers for degradation of methyl branched fatty acids (Savolainen *et al.* 2004).

### Anti-microbial Assays Reveal Anti-microbial Activity when MA is coupled to Spermine

**Figure 3:**
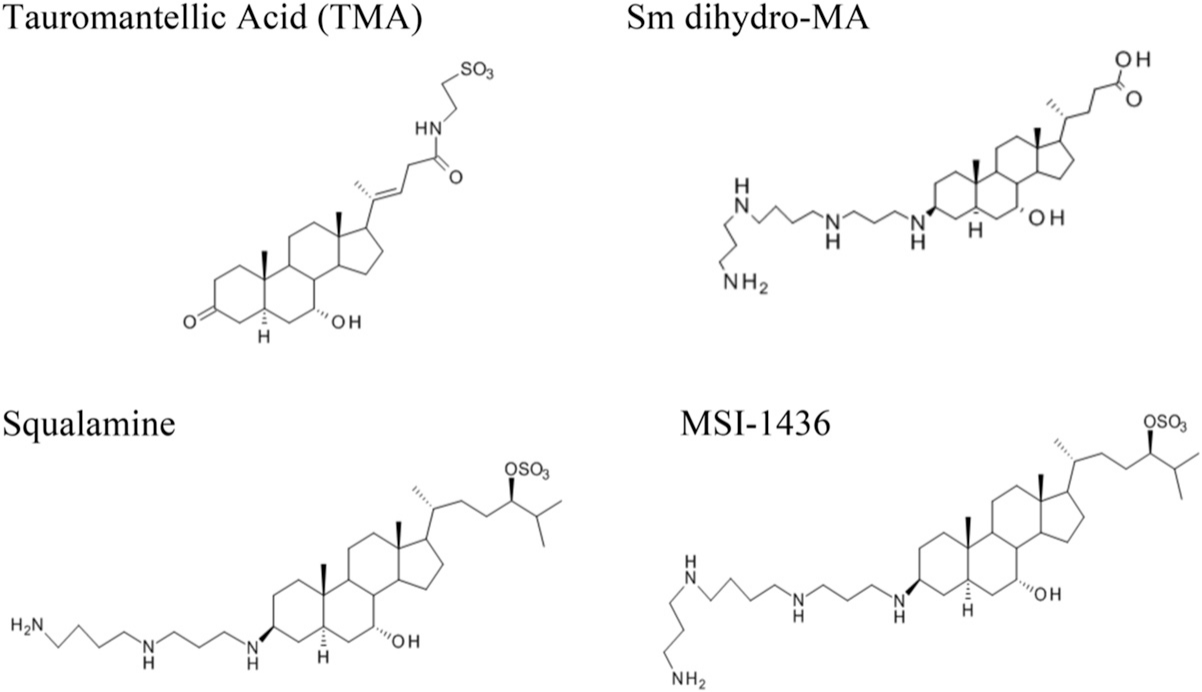
The structure of TMA, Sm dihydro-MA, squalamine, and MSI-1436 synthesized as described in Tessema *et al.* (2013). TMA was not synthesized for subsequent antimicrobial assays.

The lack of observed AMPs, the discovery of tauromantellic acid (Clark *et al.* 2012), and the functionality of the primary bile acid biosynthesis pathway in the skin of *Mantella betsileo* suggests the possibility of a distinct immunological defense system that together may constitute a broader spectrum of antimicrobial compounds. Such an immune system would require conjugating polyamines to TMA to create structurally similar molecules to squalamine, or MSI-1436 (Figure 3). We synthesized a broad-spectrum antimicrobial aminosterol (Sm dihydro-MA) by the coupling of spermine to a molecule of mantellic acid lacking the side chain unsaturation (Figure 3) and compared the activity of this molecule to other, similarly structured molecules. There was anti-microbial activity for Sm dihydro-MA, though it was not as potent as other molecules against *Staphylococcus* and *Eschericia*. However, Sm dihydro-MA was more potent against *Pseudomonas* bacteria and *Candida* fungus than ampicillin. Collectively, MSI-1436 was the most potent antibiotic against all organisms tested (Table 2).

**Table 2:** Antibiotic activity assays for Sm dihydro-MA compared with other antibiotics. The values listed are minimal inhibitory concentrations (MIC), which is the concentration of antimicrobial (μg /mL) where visible growth is inhibited. The strain’s ATCC number is noted in parentheses

	*Staphylococcus aureus* (29213)	*Eschericia coli* (25922)	*Pseudomonas aeruginosa* (27853)	*Candida albicans* (14053)
Sm dihydro-MA	16	32	16	32
Ampicillin	8	4	125	>256
Squalamine	4	4	8	8
MSI-1436	1	1	1	4

A candidate 3-keto reductase enzyme necessary to couple a polyamine to TMA for potent antibiotic activity is expressed within our *M. betsileo* transcriptome, suggesting the plausible possibility of this defense system within the skin.

## Discussion

The presence of gene-encoded antimicrobial peptides in a variety of amphibian species has fueled the perception that most frogs contain an AMP defense arsenal in their skin. These peptides are thought to be essential for protection against infectious microorganisms (but see König *et al.* 2015 for an alternative hypothesis) and are found at high expression percentiles, yet in our study we did not observe AMPs in the skin of *Mantella*. No AMPs have been found in *Mantella* previously, and there are several cases where frog lineages were examined for AMPs, yet they were not found (König *et al.* 2015). Our computational methods are based on sequence similarity and rely on available sequences and it remains possible that an entirely new class of highly divergent AMPs could be present in *Mantella* that we cannot find using computational methods. Ultimately, more careful studies of skin extracts from *Mantella*, combined with genome sequencing could help resolve whether *Mantella* have either lost or have a highly divergent class of AMPs. Nevertheless, we think they probably do not have AMPs as suggested by our studies of skin extracts from *Dendrobates*, a group of poison frogs from South America that contain alkaloid compounds and TMA. Using the same successful procedures that worked to find AMPs in other frog species (Zasloff 1987; Clark *et al.* 1994), we could not find AMPs in *Dendrobates*, yet extracts from their skin had antimicrobial activity. Collectively, this raises questions about the function and mechanistic origin of amphibian defenses, especially in lineages where AMPs may not occur.

Concomitant with the lack of observed AMPs in *Mantella* and *Dendrobates* is the presence of tauromantellic acid (TMA), which was recently discovered in poison frogs (Clark *et al.* 2012). The similarity of the ring structure of tauromantellic acid to that of the broad spectrum antimicrobial, squalamine, and the preliminary results of antimicrobial activity for mantellic acid and similar bile acids coupled to polyamines (Table 2) suggests a diversity of amphibian defenses. Tauromantellic acid is most likely synthesized from cholesterol within the skin of *Mantella* because the entire primary bile acid biosynthesis pathway is present, including the gene involved in the rate-limiting step for synthesis. The bile acid pathway members found in other frog species are most likely involved in other metabolic processes, which may account for the presence of genes in the pathway but lack of pathway functionality. Conversely, *M. betsileo* was the only species to abundantly express genes critical for functionality of the pathway including *CYP7A1*, *BAAT*, and *AMACR*.

Bile acids are detergents that play a fundamental role in both lipid and cholesterol processing mainly in the gastrointestinal and hepatobiliary systems (Smith *et al.* 2009). These chemicals are required for dietary lipid and fat-soluble vitamin absorption and maintenance of the balance between cholesterol synthesis and excretion. In general, bile acids are made primarily in the liver through the oxidation of cholesterol and their localization is tightly regulated due to their membrane and epithelial toxicity (Smith *et al.* 2009). Consequently, the fact that the bile acid pathway, which is predominantly found in the liver, appears expressed in the skin of *Mantella* is highly unusual. In humans, the presence of bile acid in the skin is associated with disease, due to high concentrations of hepatic bile acids in the blood stream.

Conversely, while AMPs are in the skin of most frogs, humans have liver-expressed antimicrobial peptides (LEAPs) that are believed to have a protective role against bacterial infection (Henriques *et al.* 2010). Thus, finding a liver associated pathway in the skin coupled with knowledge of AMP enrichment in most frogs, may suggest a deeper evolutionary connection between AMPs in the skin, genetic pathways in the liver, and potentially bile acid derived compounds used for defense that future research may uncover.

While we find moderate anti-microbial activity for MA when coupled to a spermine and the main reductase enzymes involved in conjugating spermine to MA are expressed in *Mantella*, recent discoveries suggest that bile acids may have diverse functions. Many species of fish excrete intestinal bile acids into the surrounding waters as pheromones (Buchinger *et al.* 2014), which are detected by the olfactory system and influence mating and migratory behavior. Several of the lamprey pheromones include molecules that share striking structural similarity to tauromantellic acid (Buchinger *et al.* 2015). Therefore, tauromantellic acid may play an even larger role as a pheromone or an olfactory signal.

The presence of diet-derived alkaloids, functionality of the bile acid pathway, and the lack of observed AMPs in *M. betsileo* have expanded our understanding of the versatility in potential amphibian skin defense mechanisms. Yet, the intrinsic complexity of amphibian genomes and limitations on genomic resources restrict sequencing for whole-genome assembly projects that would aid in a better understanding of the evolution of genes involved in frog skin secretions. Further analyses of neotropical poison frogs in the genera *Epipedobates* and *Dendrobates*, which also secrete tauromantellic acid, could provide new understanding of the relationship between the bile acid pathway and the complex chemical community involved in defense within anuran skin (Rogers *et al.* 2018). We predict these genera lack AMPs and have expression of the bile acid pathway in the skin. Thus, a study of poison frogs in general would serve as an intriguing direction for future research. For *Mantella* and other poison frogs, if tauromantellic acid when coupled to a polyamine provides antimicrobial functions to amphibian skin, is it interacting with alkaloids, providing antimicrobial function, or a combination of both (Raaymakers *et al.* 2017)? Additionally, are AMPs present in taxa that have evolved metabolites like tauromantellic acid, which contribute to defense, or could there be another defensive mechanism?

The synthesis of tauromantellic acid would also aid in further testing of this molecule for antimicrobial activity. In addition, a thorough chemical analysis of the skin secretions from these species of poison frogs will be needed to test the hypothesis that antimicrobial bile acids are indeed produced in their skin. Finally, generalizing the presence of 3-keto bile acids found in *Mantella* may indicate the presence of this proposed cholesterol-based immune system in other organisms, including the neonatal human (Wahlén *et al.* 1989). The novel diversity in amphibian skin defense mechanisms described in these results offers new directions for genomic research and possible applications of these immunological compounds to human health.
